# Mixed Methods Evaluation of a Youth-Friendly Clinic for Young People Living with HIV Transitioning from Pediatric Care

**DOI:** 10.3390/tropicalmed9090198

**Published:** 2024-08-28

**Authors:** Hannah Chew, Kemberlee Bonnet, David Schlundt, Nina Hill, Leslie Pierce, Aima Ahonkhai, Neerav Desai

**Affiliations:** 1 School of Medicine, Vanderbilt University, Nashville, TN 37232, USA; 2 Qualitative Research Core, Vanderbilt University Medical Center, Nashville, TN 37232, USA; kemberlee.bonnet@vanderbilt.edu (K.B.); david.schlundt@vanderbilt.edu (D.S.); 3 Department of Medicine, Section of Medicine-Pediatrics, Vanderbilt University Medical Center, Nashville, TN 37212, USA; nina.hill@vumc.org; 4 Department of Medicine, Massachusetts General Hospital, Boston, MA 02114, USA; lpierce9@mgb.org (L.P.); aahonkhai@mgh.harvard.edu (A.A.); 5 Department of Pediatrics, Vanderbilt University Medical Center, Nashville, TN 37232, USA; neerav.desai@vumc.org

**Keywords:** HIV, adolescents, young adults, transition to adult care, qualitative research

## Abstract

(1) Background: Adolescents and young adults face challenges when transitioning to adult care due to emerging adulthood and changing providers and insurance. Young people living with HIV (YPLHIV) have additional obstacles with mental health and stigma. During transition, only 55% of YPLHIV are retained in care, and 65% are virally suppressed. To address these challenges, the Adolescent and Young Adult Health Care Transition Clinic (AYAHCTC) was created at Vanderbilt University Medical Center in 2017. This mixed methods study evaluates the initial cohort and solicits YPLHIVs’ perspectives on transition barriers and facilitators. (2) Methods: Quantitative analyses (n = 21) characterized patients’ demographics, clinical engagement, and retention. Qualitative interviews (n = 5) captured patients’ transition experiences. (3) Results: This study, conducted in the Southeastern USA, included a cohort where 47.6% were born abroad, with all participants being US citizens by birth or naturalization. Patients’ mean age at first visit was 19.6 years. The average AYAHCTC duration was 2.21 years. First-year engagement and retention were 100% and 95.5%, respectively. Viral suppression rates improved from 66.7% at the first visit to 81.0% at the last visit. Eleven patients transitioned out of AYAHCTC. Qualitative analyses indicate that barriers to transition include leaving trusted providers, reduced parental guidance, developing autonomy, and perceived loss of confidentiality in adult clinic environment. Transition was facilitated by youth-friendly services, clear communication, and strong relationships with AYAHCTC providers. (4) Conclusions: YPLHIV positively viewed AYAHCTC experiences. Future directions include optimizing services to build YPLHIVs’ independence, supporting YPLHIV experiencing stigma, assuaging concerns about switching providers, collaborating with adult clinics to maintain confidentiality, and designing interventions focused on adherence during transition.

## 1. Introduction

Young people living with HIV (YPLHIV) aged 13–24 comprised approximately 41,900 individuals or about 3.5% of the 1.2 million people living with HIV in the United States in 2021 [1]. Nearly 6100 new HIV diagnoses were reported among young people during that same year, accounting for 19% of all new HIV diagnoses in the US [1]. The Southern US is disproportionally burdened with cases of HIV [2]. Interventions that can improve retention in care and adherence to treatment are essential for YPLHIV in this region, especially during their transition to adult care.

Numerous factors create challenges to transitioning YPLHIV to adult care in the Middle Tennessee Region. First and foremost, prior to 2017, there was no plan for organized and structured transition to adult care for YPLHIV at our institution. Tennessee is one of many Southeastern states that did not expand its Medicaid enrollment, and many YPLHIV struggle with knowledge and access to insurance options such as Ryan White and the insurance marketplace [3,4,5]. Pediatric and adolescent HIV care is provided within a clinic located inside the Monroe Carrell Junior Children’s Hospital at Vanderbilt (MCJCHV). Adult HIV care is provided at the Vanderbilt University Comprehensive Care Center (VCCC), which is the largest provider of HIV care in the region. The VCCC is housed at a satellite location away from the main VUMC medical campus and MCJCHV. Although these two locations are only 8 miles apart, this physical relocation represents a barrier for YPLHIV and their families, who may be unfamiliar with the area. Finally, the population of YPLHIV in the region is very diverse, including those with perinatally acquired HIV (PAH) and those with non-perinatally acquired HIV (NPAH) [6,7]. PAH can be further divided into domestically born or internationally born individuals. Internationally born individuals could be refugees or international adoptees [8,9]. Among NPAH, there is diversity in sexuality and gender identity [10]. Given their heterogeneous backgrounds, developing a transition program requires a great deal of flexibility and individualization.

While a growing number of studies detail interventions to help YPLHIV transition to adult care, few elicit feedback and input from YPLHIV about the barriers and facilitators to successful transition [11,12,13,14,15]. There is the robust literature supporting the involvement of YPLHIV in all aspects of design, implementation, evaluation, and modification of services which are intended for them [16,17]. Although research from other regions, such as sub-Saharan Africa, has provided valuable insights, the distinct sociocultural and healthcare challenges in the Southeastern US, where HIV-related health disparities are especially pronounced, highlight the need for continued evaluation of transition interventions [13,18,19,20,21]. It is critical to obtain feedback from the receiving YPLHIV to enhance the services that are offered during the transition to adult care.

This study aims to evaluate quantitative data, including demographics, engagement in care, retention in care, and viral suppression of the initial cohort of patients in the Adolescent and Young Adult Health Care Transition Clinic (AYAHCTC). In addition, this study elicits qualitative information about facilitators and barriers to transitioning to adult care from the perspectives of YPLHIV. This study aims to build understanding of optimal interventions for YPLHIV’s transition and use patients’ feedback to guide the future direction of services provided.

## 2. Materials and Methods

### 2.1. Overview

This mixed methods study includes data collection from two populations. The first is the entire cohort of AYAHCTC attendees who contributed quantitative demographic and clinical data. The second is a subset of AYAHCTC attendees who contributed qualitative data collection in the form of semi-structured in-depth interviews (capturing patients’ experiences at AYAHCTC and their transition between care settings) in addition to psychosocial measures that may have influenced their transitions of care.

### 2.2. Setting

The Adolescent and Young Adult Health Care Transition Clinic (AYAHCTC) was created at Vanderbilt University Medical Center in Nashville, TN, USA, in 2017 to address the challenge of transitioning YPLHIV to adult care. This intervention consists of a youth-friendly clinic that encompasses primary care, HIV-related care, medical case management, and healthcare transition navigation. The multidisciplinary AYAHCTC team consists of physicians, a social worker, a nurse case manager, and a healthcare navigator. Patients enter the AYAHCTC from Vanderbilt’s Pediatric HIV Clinic, other pediatric clinics, or community referrals (including testing sites). Patients later transition from the AYAHCTC to the VCCC.

### 2.3. Participants/Recruitment

Inclusion criteria for this study were any individual 18 years and older who had received care at the AYAHCTC since 2017 and who had consented to be contacted for future research. All patients had an existing provider relationship with the principal investigator (ND). The principal investigator recruited patients for the interview through text and direct-to-patient messaging on a secure, online patient portal. Informed consent was obtained from patients who agreed to participate in the interview. Informed consent clearly stated that participation in this project would not affect clinical care and that individual responses would be kept confidential from any AYAHCTC care providers. This study was approved by the Vanderbilt University Medical Center IRB #231140.

### 2.4. Quantitative Data Collection Procedures

For the entire clinic cohort, patient health information was extracted from EPIC using a standardized data collection tool including appointment dates, HIV viral loads, CD4 counts, and basic demographic data including age, gender, self-identified ethnicity, and HIV acquisition mode. Engagement in care was evaluated according to the CDC definition, as the percentage of patients attending at least one HIV medical care visit in the observed year and retention in care is defined as the percentage of patients who had at least two CD4 or viral load tests obtained at least three months apart. Viral suppression was measured as the most recent viral load being less than 200 [22].

### 2.5. Semi-Structured Interviews

We conducted semi-structured in-depth interviews with a subset of clinic attendees. The first part of the interview consisted of structured questions from validated tools, such as the (1) Social Provision Scale [23,24,25], (2) Brief 12-Item HIV Stigma Scale [26], (3) Patient Health Questionnaire [27], and (4) Transition Readiness Assessment Questionnaire [28]. The second part of the interview was a formal in-depth interview with interview guide questions and probes (Supplement File S1), focused on evaluating the patient’s experiences related to the AYAHCTC clinical areas, staff, services, as well as facilitators and barriers to transition between care settings.

A team member (HC, female, medical student) trained in qualitative interviewing recorded online video interviews lasting 45 min using a HIPAA-compliant institutional Zoom account meeting data security requirements. To minimize risks to participants, the interview files were de-identified. The original audio and video recordings of interviews and survey tools, which excluded participant names, were uploaded in a HIPAA-compliant database. Audio files were submitted to an IRB-approved transcription service (rev.com (accessed on 24 April 2024)). Patients were compensated with a USD 50 Uber gift card for completing the interview.

### 2.6. Qualitative Analysis

Qualitative data coding and analysis was conducted by following the Consolidated Criteria for Reporting Qualitative Research (COREQ) guidelines, an evidence-based qualitative methodology [29]. A hierarchical coding system (Supplement File S2) was developed and refined by HC and KB using the interview guide and a review of the transcripts. Major categories included (1) “Specific elements of the clinic for quantitative ratings”; (2) “Ratings”; (3) “Cognitions”; (4) “People”; (5) “Barriers and facilitators”; (6) “Communication”; (7) “Services”; (8) “Transition”; (9) “Emotion”; (10) “Needs and suggestions”; (11) “Change over time”; (12) “Notable quotes”; (13) “Other clinic sites”; (14) “No change over time”; and (15) “Knowledge and understanding of HIV status or disclosure”. Major categories were further divided from one to 10 subcategories, with some subcategories having additional hierarchical levels. Definitions and rules were established for each category and subcategory.

HC and KB independently applied codes to the first two transcripts and jointly reconciled discrepancies to establish inter-rater reliability. HC then coded the remaining three transcripts. Each statement was treated as a separate quotation and could be assigned up to 19 different codes. Transcripts were combined and sorted by code. Transcripts, quotations, and codes were managed using Microsoft Excel 2016 and SPSS version 28.

We used an iterative inductive/deductive approach to qualitative data analysis [30,31,32]. Inductively, we sorted the coded quotes by coding category to identify themes and relationships among themes. Deductively, we were guided by the health systems science framework, as it helped us to understand the structures and processes involved in care transition [33]. We were also guided by the biopsychosocial model, as it helped us to understand the dynamic interplay between biological, psychological, and social factors that influenced patients’ experiences within the health system [34,35]. The process was iterative in that the framework was informed by theory, while specific content in the framework was derived from the qualitative data.

## 3. Results

### 3.1. Description of Sample

Patient health information for the study’s descriptive analyses was collected from all 21 eligible patients. All eligible patients were invited to participate in the qualitative interview, of which five (n = 5) completed both the consent process and interview. The interviewed patients all identified as Black males and ranged between 18 and 30 years with a mean of 23.8 years. Table 1 summarizes the eligible AYAHCTC patient population’s and the interview patients’ demographics. Table 2 reports markers of eligible patients’ clinical engagement at AYAHCTC. Viral suppression at adolescents’ first AYAHCTC visit was 66.7%, compared to 81.0% at their last visit. Eleven of the 21 patients (52.4%) transitioned to adult care at the VCCC.

Table 3 details responses (n = 5) from the survey’s various validated scales measuring social provision, stigma, transition readiness, and depression. The Social Provision Scale is scored between 4 and 16, where a high score indicates that an individual is receiving provision. The mean score overall was 14.1, whereas subdomain scores were the following: “Guidance” (15.6), “Reliable Alliance” (15), “Social Integration” (14.4), “Reassurance of Worth” (14), “Attachment” (14.2), and “Nurturance” (11.6). The HIV Stigma Scale, which ranges from 3 to 12 with higher scores indicating higher perceived stigma, revealed an overall mean score of 6.7. It also reported means in different domains: “Personalized Stigma” (4.2), “Disclosure Concerns” (11.0), “Concerns about Public Attitudes” (6.8), and “Negative Self-Image” (4.6). The mean self-assessed medication adherence on a scale of 0 to 100%, where 100% means complete medication adherence over the past 30 days, was 88.6%. The Transition Readiness Assessment Questionnaire is scored between 1 and 5, where a higher score represents greater levels of transition readiness. The overall mean score was 4.3. The mean scores in various domains were the following: “Managing Medications” (3.96), “Appointment Keeping” (4.35), “Tracking Health Issues” (4.27), “Talking with Providers” (4.64), “Motivation” (4.60), and “Self-Efficacy” (4.20). The Patient Health Questionnaire-9 is scored between 0 and 27, where a higher score represents a higher severity of depression. The mean was 2.0 and ranged between 1 and 11. Statistical analysis of the standardized questionnaire data was not possible due to the small number of respondents.

### 3.2. Framework for Patients’ Perceptions of Factors Affecting Transition from Adolescent to Adult Care

**Figure 1:**
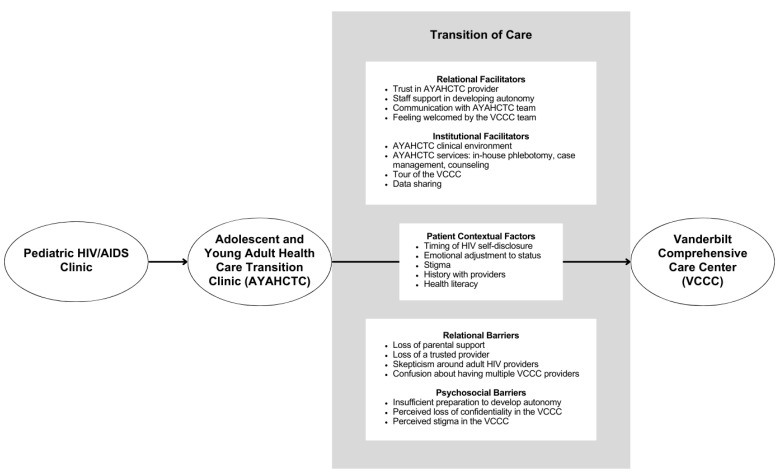
Framework for Patients’ Perceptions of Factors Affecting Transition from Adolescent to Adult Care.

Figure 1 presents the conceptual framework of patients’ perceptions of factors affecting the success of their transition from adolescent care at AYAHCTC to adult HIV care at the VCCC. The conceptual framework was derived from thematic analysis of qualitative data from the interviews. The figure also displays the three different settings where patients could receive HIV care based on their developmental stage.

The box directly between “AYAHCTC” and “VCCC” details self-identified patient-specific factors that affected their transition between care settings. The uppermost box summarizes patients’ perceived facilitators of transition, which was further categorized into relational and institutional facilitators. Relational facilitators of transition involved patients’ relationships with providers and clinic staff, whereas institutional facilitators encompassed organized clinic services and protocols. The lowermost box between “AYAHCTC” and “VCCC” illustrates patients’ perceived barriers to transition, which was further subdivided into relational and psychosocial barriers. Relational barriers of transition represented patients’ relationships with providers and clinic staff, whereas psychosocial barriers delineated the patients’ cognitions related to transition. In the following sections, we will discuss each component of the conceptual framework and illustrate the discussion with quotes from patients.

### 3.3. Major Themes

The major themes from the qualitative analysis are each presented as a table, and the subthemes are represented within each table and supported by direct quotations from patients.

#### 3.3.1. Theme 1: Patient Contextual Factors

Qualitative analysis of interviews revealed patient contextual factors (Table 4), where patients identified factors specific to themselves that affected their transition between care settings. The subthemes were (1) timing of HIV self-disclosure; (2) emotional adjustment to HIV status; (3) stigma; (4) history with adolescent care providers; and (5) health literacy.

Timing of HIV self-disclosure—Some patients commented on how their age of diagnosis affected their engagement with AYAHCTC and the healthcare system. For one PAH patient, disclosure of HIV status did not have a significant impact on their transition because “I’ve always been taking medication […] I never knew why I was taking medication, until I turned maybe 10 and that’s when they told me. So, at that point, it was just like, ‘Okay.’ It was never really a struggle living with HIV. […] It was just something I lived with”.

Emotional adjustment to HIV status—Some patients also described their emotional adjustment to HIV status after disclosure. For one patient, “It took years. It was just years of conversation from the adolescent clinic, from my provider that was at my children’s hospital. […] And when I say years, years, not two or three, this is like 10 years of conversations trying to get the guts and the courage to sit and talk about [my HIV status] and stop leaving it sitting in on me and it stressing me out. And it’s a lot of buildup, anger and stuff like that”.

Stigma—All patients discussed their experience with stigma surrounding their HIV status. Patients mentioned stigma in several contexts, such as at AYAHCTC, VCCC, school, and in social circles. One patient mentioned sometimes he overall was “Just feeling like an outcast”. This same patient was uncomfortable saying his diagnosis aloud even in the privacy of his own home, as he disclosed, “I don’t like saying [HIV], so you got to, excuse me. I’ll probably say ‘it’ or ‘status’”. When discussing the AYAHCTC front desk staff, one patient remarked, “I guess dealing with that status, you kind of wonder if some people are really nice because they feel bad or they’re just being nice because they’re nice people. But I always like to think that they would be nice because they’re nice people, and that’s how I look at it”.

History with providers—All patients shared similarly strong relationships with their AYAHCTC provider, often citing their trust in providers. One patient commented, “I still keep in touch with [Dr. NAME at AYAHCTC] […] Very friendly staff, physicians very friendly, just makes you feel like you don’t have HIV to begin with”.

Health literacy—Finally, some patients shared how their health literacy impacted their understanding of HIV diagnosis and management. Patients exhibited variable health literacy, most notably surrounding their laboratory results. For example, one patient shared, “The [laboratory] results, I personally don’t ever really check on until I go back to the office […] there’s a lot of things that I don’t want to worry myself about and they’re actually normal. So, I don’t even bother looking at my test results on my own, but I know that if I were, [Dr. NAME] is going to explain them to me, he’s going to explain them very thoroughly, he’s going to make an action plan with me right then and right there”.

#### 3.3.2. Theme 2: Transition of Care Facilitators

Patients described the perceived facilitators of their transition from adolescent to adult HIV care (Table 5), citing relational facilitators, including (1) trust in their AYAHCTC provider; (2) staff support in developing autonomy; (3) communication with their AYAHCTC team; (4) feeling welcomed by the VCCC team; and institutional facilitators including (5) the AYAHCTC clinical environment, (6) AYAHCTC services, such as in-house phlebotomy, case management, and counseling, (7) a tour of the VCCC, (8) the first VCCC appointment scheduled for patients, and (9) data sharing.

##### Relational Facilitators

Trust in AYAHCTC provider—All patients expressed high praise for their doctors at AYAHCTC. They described their providers as patient, compassionate, and helpful. All said they would recommend their provider to a peer. One patient’s quote exemplifies this: “[Dr. NAME] is very informative. He doesn’t treat you like any other doctor does, where they just talk over you and explain things that you may not necessarily know. I like the fact that [Dr. NAME] actually is there for his patients”. Patients also felt more comfortable in their transition to adult care by trusting their AYAHCTC provider’s recommendation of a new provider. For example, one patient stated, “And then the adolescents, [Dr. NAME at AYAHCTC] already knew [Dr. NAME at VCCC]. So, the fact that the two communicated made me more accepting of the transition”.

Staff support in developing autonomy—Patients noted that AYAHCTC staff empowered them to take a larger role in their own HIV care and helped build skills to become more independent. One patient said, “My parents used to schedule all the appointments for me, but now before fully transitioning, [my providers] basically taught me, ‘Hey, you need to start doing this’, gave a little more responsibility, a real small step until I was fully able to do appointments by myself, order prescriptions myself. So, they slowly over a couple months or so were like ‘Hey, I’m going to start slowly not doing things for you’, and you slowly start picking up your own medication, which I got used to really quickly. And after that I was like, okay, I’ve been doing this for quite a while, I know what to do now”.

Communication with AYAHCTC team—Most patients agreed that communication with AYAHCTC staff was easy and reliable via phone calls and MyHealth at Vanderbilt. One patient said, “If I need something after the appointment that I forgot to talk to [Dr. NAME] about, I can ask my nurse and they’ll go get him. Or if he’s in another patient’s room, he’ll tell me, ‘Well, text me on the telehealth app […]’ He’s always there”. Some patients explicitly appreciated that their transition process felt gradual because the provider communicated to the patient early what the next steps in their care entailed. One patient said, “In the adolescent workup, yes, they prepared me for the next step, be like, ‘Hey, for the next step you’ll have to do X, Y, Z by yourself with little help from your parents’, which mentally prepared me like, okay, this is all for me and my health matters to me and my parents can intervene if need be, but this is all for me. So, it helped me get ready”.

Feeling welcomed by the VCCC team—Some patients met their adult HIV care provider during the transition process, either as part of the VCCC tour, or as a separate meeting. Those who had this opportunity remarked that they felt accepted, “I met the physician that was going to be helping or taking care of me. I met the caseworkers, case managers, the front desk people. And it was more of a relief that everybody was very friendly towards [Dr. NAME from AYAHCTC] and they knew the whole situation. So, I guess it was relieving, not too scary”.

##### Institutional Facilitators

AYAHCTC clinical environment—Patients overall felt positively towards AYAHCTC staff and the physical space. Many patients said that the front desk staff were friendly and helpful. Some patients also commented that they did not worry about unwanted HIV status disclosure in the waiting room environment since all adolescents, regardless of HIV status, waited there. One patient emphasized this, stating, “People are not staring at me. They don’t know why I’m going there because, I mean, it’s just an adolescent clinic, so it’s not like no one knows, oh, he has HIV”. Patients also acknowledged that being in a hospital could sometimes be intimidating, but that the examination room was generally a comfortable environment. One patient specifically liked the privacy curtain because “I didn’t always feel like at any given moment, somebody could walk in, and I’d be exposed”.

In-house phlebotomy—The in-house phlebotomy room and laboratory technologists were viewed positively by patients. Many found that they were calmed by decorations in the phlebotomy room and appreciated when laboratory technologists talked to them and calmed them during routine blood draws. One patient found that they were a valuable resource in understanding their care, “And if I had a curious thought about why, what was the blood for, what’s the purpose of me getting the blood draw, they would tell me to get sample of this diagnosis, whatnot, and I’ll be at ease at that moment knowing that”. Patients liked that the AYAHCTC phlebotomy room was in the same clinical area as their examination room.

Case management/healthcare navigator service—Patients generally felt that the case management service was helpful. One patient noted that “The [caseworker] will send me information on insurance policy plans, new prescriptions, and health plans. So, he’ll just keep me updated with new information surrounding about the healthcare of HIV, new medication, new testing that’s going on. He’ll text me at least once a month, making sure I’m good. So, he was a very friendly guy”. However, that same patient also mentioned that he initially felt uncomfortable disclosing his HIV status to a new team member because “It’s like, I don’t even know who you are. You haven’t been around. And I’m not even comfortable with telling close people. How do you know? It just didn’t feel comfortable for me to talk to them”.

Counseling service—Only two of the patients interacted with AYAHCTC’s counselors, who offer psychological and emotional support specifically for this patient population. One patient found that the AYAHCTC counselor was helpful and even provided additional mental healthcare recommendations. However, the other patient discontinued his visits with a counselor because “I didn’t like talking to people that hadn’t been through the same things and understood exactly what I was going through. It just felt very weird, and in a way, I felt a little judged going to someone”. Other patients did not utilize this service because they either lacked the need for extra support or had therapy external to AYAHCTC.

Tour of the VCCC—Providers organize a tour of the VCCC prior to patients’ final appointment at AYAHCTC. Overall, patients who agreed to the tour felt positively after meeting new staff and providers and previewing the clinical environment. One patient elaborated, “[Dr. NAME] set up a whole tour with somebody who was in the adult care […] Let me get to know the people who are in the adult clinic, so the transition would be a little bit easier. So, that was very helpful to me, as far as getting to know where I’m going, how it looks, instead of just being pushed into the unknown”.

Data sharing—Finally, some patients acknowledged that AYAHCTC providers transferred their patient files, including notes, medication history, and laboratory results, to VCCC providers, making the transition process easier. One patient noted that his medical records were transferred from the adolescent clinic to the adult clinic, which made him feel recognized and contributed to a positive experience during the transition period. “They already knew who I was and stuff when I got there […] I guess they transferred my file or something, but I don’t know”.

#### 3.3.3. Theme 3: Transition of Care Barriers

Patients’ perceived barriers to the transition of care from adolescent to adult HIV care (Table 6) emerged as a major theme. These barriers encompass relational and psychosocial factors. Relational barriers included (1) loss of parental support; (2) loss of a trusted provider; (3) skepticism about adult HIV providers; and (4) confusion about having multiple providers. Psychosocial barriers included (5) insufficient preparation to develop autonomy; (6) loss of confidentiality in the VCCC; and (7) perceived stigma in the VCCC.

##### Relational Barriers

Loss of parental support—Some of the patients attended all their appointments at the AYAHCTC with at least one of their parents. One patient experienced a noticeable difference in how he felt at his first adult HIV care appointment, as “It was weird in a way since I’ve been only going with my parents and whatnot, so it was all up to me to listen and get the information and process it and choose the next option. So, I was usually the one to sit back and twiddle my thumbs while the parents talked to the doctor. But now just I was forced […] to listen and hear instead of playing around”.

Loss of a trusted provider—All patients had been at AYAHCTC for multiple years and developed a strong relationship with their physician. This trust and companionship made it harder for them to accept leaving AYAHCTC for a new provider and clinic. One patient described this: “For me, it’s not the fact that I have to transfer, it’s more so the fact that I have to have a new doctor, and I feel like I’ve built a bond with [Dr. NAME] already, and that what we have going on is something that’s working for us. And I really like that he’s starting to understand me, I’m starting to understand his expectations a lot more, and he just cares about his patients, so I want to see him [at the adult clinic]”.

Skepticism around adult HIV providers—Patients often had preconceived notions about adult HIV care providers that added another obstacle to transition. For instance, a patient explained, “I feel like a lot of adult doctors, they’re just there just to examine you and go on about their day. I like the conversations that me and [Dr. NAME at AYAHCTC] have, because sometimes I do go in there nervous, because I have very bad anxiety that I’m trying to work on, and he understands that. So, he talks to me, he explains everything. I feel like I’m not going to get that at other places at all”.

Confusion about having multiple VCCC providers—Patients did not have a point person at the VCCC like they did at AYAHCTC. One patient compared the two: “[Dr. NAME at AYAHCTC] has my personal number, so we communicate through there. […] They’re switching out my [adult care] physician, so I never know what new physician I have. It’s usually on MyHealth”.

##### Psychosocial Barriers

Insufficient preparation to develop autonomy—Some patients felt unprepared to handle their own healthcare independently and wished they had built more skills prior to completing their clinic transition. One patient expressed his apprehension, stating, “I guess the beginning of the adult [care] […] just taught me you are responsible for your health and not anyone else. And that came as a shock in a way because I always relied on others to make choices for my health, but the fact that I’m in charge of making it is scary”.

Loss of confidentiality in the VCCC—Patients expressed reluctance to transition to the VCCC, due to their perceived lack of confidentiality in the waiting room. The VCCC is specifically designed for adults living with HIV, so everyone in the waiting room is aware of other people’s HIV status. The AYAHCTC is housed in the adolescent clinic, where patients are seen for a variety of general health needs. One patient attributed his long transition in care to the loss of privacy: “When I was a kid, it was okay because everybody was in [AYAHCTC] for different reasons. But as an adult, it’s just kind of like, I don’t know, very uncomfortable because [CCC is] in a whole separate part of the building, and it’s isolated. […] And everybody’s in there for the same thing. So, I feel like that takes the privacy away. So, I didn’t like that part. And that’s what took me so long to get out of the children’s part”.

Perceived stigma in the VCCC—Patients expressed concerns about being stigmatized by peers if seen in the VCCC. One patient expressed his fear of judgment from peers at school, as he elaborated, “But with the CCC, you go in there and I went to school with this person and there’s no other reason for you to be checking in over here but for that and I don’t know, it just felt weird. Now it’s one of those things where rumors can start, people accidentally saying something, ‘Oh, I seen such… How did you see him when you…’ So, all that, it’s just so uncomfortable”.

## 4. Discussion

Successful transition to adult care for YPLHIV requires proficiency in seeking medical assistance, scheduling appointments, accessing prescriptions, and managing health insurance, alongside specific considerations related to HIV management [36]. However, at this stage, adolescents are still developing executive function, impacting YPLHIV’s ability to navigate complex healthcare systems, adhere to treatment, and anticipate long-term health outcomes [37,38]. Although establishing autonomy is a natural part of late adolescence and young adulthood, the demands of managing a chronic condition with daily medications and regular appointments can often leave them feeling disempowered [39]. Additionally, YPLHIV may struggle to disclose their status to peers given the importance of social acceptance during adolescence. Similarly, exploring sexual identity during this age is further complicated when having to navigate partner disclosure [40]. Addressing these developmental needs is essential for designing effective interventions that support YPLHIV in their transition to adult care.

In addition to developmental challenges, YPLHIV face obstacles with stigma, discrimination, and internalized fears or mistrust of medical systems [41,42,43,44,45]. They also face complex challenges such as mental health disorders, substance use, poverty, family trauma, and societal pressures [46,47]. This study highlights some barriers to successful transition which include insufficient self-efficacy, issues with healthcare providers, and disclosure issues. It also highlights facilitators to successful transition, including youth-friendly services, effective communication, and the importance of personal relationships.

### 4.1. Patient-Identified Barriers to Transition of Care

An important barrier emphasized by our patients can be related to self-efficacy. In this context, self-efficacy is defined as “the extent or strength of one’s belief in one’s own ability to complete tasks and attain goals despite environmental and social barriers” [48]. Health literacy is essential for fostering self-efficacy, as it empowers patients to make informed decisions about their care. Health literacy was brought up as an issue for some of the patients. Viral suppression improved across AYAHCTC visits, and patients self-reported 88.6% medication adherence over the past 30 days. Still, some patients did not understand the role of medicines. The TRAQ questionnaire further highlights this, showing patients felt least prepared in managing their medications. This may also explain the marginal improvement in viral suppression rates.

Low self-efficacy is also associated with insufficient autonomy. Patients felt less confident in managing their own care as they lost parental support surrounding their HIV care. Patients commented that they were shocked at their lack of preparation for transition, which contrasts with patients’ high average rating of current self-efficacy as 4.2 out of 5 on TRAQ. Qualitative comments reflect initial challenges during the transition, but the TRAQ scores of patients who completed transition demonstrate that patients now feel more self-efficacious. This highlights a significant growth in their confidence over time, emphasizing the value of ongoing support and experience in fostering self-efficacy. Patients’ high mean score of 4.6 in the motivation domain of TRAQ illustrates their strong desire to independently manage their own healthcare. This is further reflected by the high clinic’s engagement and retention in care. By leveraging patients’ intrinsic motivation, we can implement these strategies more effectively to improve other transition outcomes, such as viral suppression.

Patient feedback was instrumental in understanding the significance of provider-related transition barriers. For some patients, the loss of a trusted provider at AYAHCTC emerged as a major barrier to transition, highlighting the strong positive relationships they had built with the clinic’s providers. These concerns are similar to other qualitative studies which found that YPLHIV view adolescent providers as “family” [49,50]. Patients also expressed skepticism and apprehension about adult HIV care providers, citing concerns about receiving personalized care and support comparable to their experiences at AYAHCTC. Preconceived notions about adult care settings as impersonal and transactional further contributed to patients’ reluctance to transition. Targeted interventions, such as training adult HIV care teams in youth-friendly services, are needed to address misconceptions and promote trust in adult care providers.

Some patients experienced confusion about having multiple new providers at the VCCC. They expressed a desire for AYAHCTC and VCCC providers to designate a point person at each clinic to facilitate continuity of care. The lack of a familiar face and personalized support at the VCCC contributed to feelings of disorientation and apprehension. Although few clinics have transition-specific staff, one study had unanimous agreement among providers that there was utility in a social worker or case manager who could serve as a dedicated transition coordinator [51]. Some HIV care teams found the value of internal medicine/pediatrics-trained providers who could continue to manage the YPLHIV who had transitioned to adult care [52].

Some PAH patients articulated challenges associated with the emotional adjustment to their HIV status following disclosure. Disclosure of HIV status from healthcare teams and guardians is a complex process for children with PAH [53]. The World Health Organization (WHO) recommends that disclosure of status occurs incrementally, starting at younger ages, with full disclosure by age 12 [54]. It often takes young teens years to process this full disclosure and understand the ramifications within their family and in society. Patients reflected on the age at which they received their HIV diagnosis and its implications for their social interactions and engagement with healthcare services. Those who had been aware of their HIV status from a young age described mixed concerns around stigma from loved ones and healthcare workers.

Disclosure is also applicable to youth with NPAH. One NPAH patient who was diagnosed in his teenage years described a depressive period after disclosure but sought counseling early on. A few studies have found that NPAH youth report more depression, anxiety, and internalized stigma [55]. Varied reactions to disclosure underscore the importance of the ongoing assessment of adolescents’ individual psychological needs and offering support and guidance to facilitate emotional adjustment following their disclosure of HIV status [56,57].

Embedded within the qualitative feedback from our patients was a collective fear of successive disclosure to new healthcare team members. This included hesitancy to work with counselors, case managers, and healthcare navigators who could help the young person prepare and actuate transition of care and maintain adherence to treatment. This theme of fear of successive disclosure was embodied by their preference for in-clinic phlebotomy services at AYAHCTC, where they encountered the same phlebotomist at every visit. In contrast, when a provider orders labs at the VCCC, the patient must go down to the main lab, which involves a different phlebotomist each time. As such, a new person becomes aware of the patients’ HIV status with each of those lab draws. The patients also noted that the transition to the VCCC involved meeting new people, such as providers, social workers, case managers, and ancillary staff. Combined, these successive disclosures can have a powerful effect of delaying or impeding the transition to adult care.

YPLHIV also fear inadvertent disclosure to non-medical persons during their transition to adult care. Since the AYAHCTC operates within a general AYA clinic, patients waiting in the lobby are unaware of each other’s HIV status, alleviating concerns about inadvertent disclosure and fostering feelings of security and privacy. In contrast, some patients perceived reduced privacy and heightened visibility in the VCCC, a dedicated space for adult HIV care. As a result of their concerns regarding confidentiality breaches, patients increasingly felt the effects of stigma related to HIV, making it more challenging to successfully transition out of AYAHCTC. All of this was corroborated by the fact that all five patients scored high in the “Disclosure to Others” category of the HIV Stigma Scale (mean = 11, range 10–12). This contrasts with lesser concerns about how loved ones and the public viewed their status, as indicated by lower scores in the “Personalized Stigma” (mean = 4.2) and “Concerns about Public Attitudes” (mean = 6.8) domains. Moreover, the relatively low score in the “Negative Self-Image” domain (mean = 4.6) combined with low PHQ depression scores suggest that while internalized stigma and self-perception may not be major issues in patients’ psychological well-being, the fear of inadvertent disclosure remains a significant barrier to transitioning into a clinic exclusively for adults living with HIV.

### 4.2. Patient-Identified Facilitators to Transition of Care

Patients universally highlighted the importance of youth-friendly services as a helpful tool for building confidence to transition. For example, patients provided overall positive feedback for AYAHCTC’s case management and healthcare navigator service, which helped coordinate care plans, address social needs, and provide additional education relating to HIV. Counseling services further addressed patients’ psychosocial needs. Patients expressed satisfaction with AYAHCTC’s clinical environment, highlighting the comfortable atmosphere and friendly staff. Providing youth-friendly services and an environment welcoming to YPLHIV may have contributed to high engagement and retention in care.

Another example of a youth-friendly service was the idea of a warm handoff between providers in the AYAHCTC and the VCCC. Warm handoffs are defined as a transfer of care conducted in-person between the adolescent and adult providers, in front of the patient [58]. Warm handoffs have a strong base of evidence in transitions of care [59,60]. Patients appreciated the tours of the VCCC, which were set up and conducted by a social worker or case manager from the AYAHCTC. The tours provided a low-pressure opportunity to familiarize patients with the new clinical environment and their future healthcare provider team at the VCCC. A future intervention to improve the youth-friendliness of the VCCC would be to implement in-clinic phlebotomy services, given their significance in satisfying AYAHCTC patients.

Our respondents stressed the importance of early and effective communication with patients and among care providers. Providers discussed how transition to adult care would progress at the very first AYAHCTC visit. Patients appreciated this early discussion because it gave them more time to process and prepare for the next steps. They were also very appreciative of the number of ways that they could communicate with their healthcare team including texts, phone calls, and the secure patient health application MyHealth@Vanderbilt. Patients’ high accessibility to AYAHCTC providers likely prepared them to effectively communicate their needs with future healthcare providers, as reflected by a high mean TRAQ score of 4.64 out of 5 in the “Talking with Providers” domain. Healthcare team members received specialized training to use communication methods that avoid explicitly labeling or disclosing the HIV status of patients, thus mitigating risk for unintentional disclosure to others. The use of mobile-based technology has shown to be feasible and acceptable for YPLHIV [61,62,63].

One important part of communication was among AYAHCTC and VCCC providers about the transfer process. Patients appreciated that there had been communication about their preferences, medical/social history, and priorities among the providers. The transfer of patient records, including notes, medication histories, and laboratory results, between AYAHCTC and VCCC providers was recognized as a facilitator of transition. Inter-clinic communication and data sharing facilitated informed decision-making and personalized, continuous care in the adult care setting [64]. This aligns with the Health and Human Services Panel on Antiretroviral Guidelines for Adults and Adolescents with HIV, which suggests regular meetings and case conferences to facilitate consistent conversations between adolescent and adult teams before and after transition [36].

Patients clearly emphasized the value of personal relationships as they were transitioning to adult care. They valued the strong bond that they had with their pediatric and AYAHCTC providers and trusted their opinions and recommendations about providers at the VCCC. They also trusted that the AYAHCTC providers had their best interest at heart, which is further reflected in the Social Provision Scale scores, where the highest scores were in the domains of “Guidance” (mean = 15.6) and “Reliable Alliance” (mean = 15). Trust in healthcare providers can be leveraged to improve transition outcomes. Trust and comfort are important aspects of other transition programs, such as the Paseo program in Peru, which provisions a community-based accompaniment during the transfer to adult HIV care [65,66]. The idea that personal relationships are important underscores the importance of training adult providers to be welcoming and understanding when patients initially enter the adult clinic. A positive first impression will help lay the foundation for fostering trust in the patient–provider relationship.

### 4.3. Limitations

This study has several key limitations. The study was conducted at a single site, which may have limited its external validity and generalizability to other transition programs for YPLHIV with different patient populations, clinic structures, and transition practices. Secondly, the participation rate of only five out of 21 members raises concerns about selection bias. YPLHIV who chose to participate may have been more engaged with their care, potentially biasing the results towards individuals who were more likely to be virally suppressed and retained in care. This selection bias may have caused an underestimation of the barriers to successful retention, adherence, and transition for this cohort and beyond. Since such a small number of individuals participated in the qualitative interviews, any interpretations of the standardized validated tools such as the SPS and TRAQ are severely limited. Another limitation is that interviews were conducted online, which may have been unable to capture non-verbal cues, such as body language, which are more easily observed in face-to-face interactions. Virtual interviews may reduce barriers to participation, such as transportation and time constraints, but they may also introduce challenges, including discomfort for participants who are unable to find a private setting conducive to honest communication about sensitive topics. Although viral suppression improved during the patients’ time at the AYAHCTC, suppression was still not ideal (>90%). This underscores a major limitation that despite supportive interventions during transition of care, viral suppression was only marginally improved. Finally, participatory research poses challenges due to the stigma associated with HIV status disclosure, which may inhibit patients from openly discussing their experiences and perspectives, especially with researchers or unfamiliar people.

### 4.4. Future Directions

Several future directions were identified from this research. First and foremost is inclusion of YPLHIV in the entire process beginning with needs assessment, program development, implementation, and evaluation [16]. A Youth Advisory Board (YAB) was utilized at various stages of this project. The utility of the YAB was limited by concerns about confidentiality and HIV status disclosure among participants. A potential solution is the development of a neutral HIV status peer support group in which HIV status is not the focal point of the membership. The YAB was also limited to online meetings due to the COVID-19 pandemic. Another future initiative is implementing peer groups and peer transition champions to provide psychosocial support for YPLHIV as they adjust to their HIV diagnosis and management and transition to adult care [67]. Peer groups may also be valuable in supporting YPLHIV with challenges around stigma. Early peer engagement has been shown to be effective for improving viral suppression, retention, and engagement in care, as well as antiretroviral adherence throughout the continuum of HIV care [68,69]. Feedback from YPLHIV also underscored the utility of transition protocols in facilitating a smoother process. Such protocols should streamline early communication about the transition process, build skills needed to manage their HIV care independently, and ensure warm handoffs between pediatric and adult care providers. Protocols may encourage patients and their families to be more proactive in their transition and better delineate each staff member’s responsibilities [12,14]. Furthermore, in-house services, such as phlebotomy, may reduce the need for disclosure. Finally, staff trained to address adolescent needs may overall create a more comfortable and welcoming environment. These strategies may better address the unique needs of YPLHIV and improve health outcomes.

## 5. Conclusions

The findings of this study underscore the value of centering the feedback of YPLHIV in the design and implementation of programs directed at improving HIV care transitions. The positive experiences reported by patients highlight the success of AYAHCTC in meeting the unique needs of YPLHIV. Interventions to improve medication adherence and viral suppression are still needed. Future studies are needed to evaluate these results in other clinic settings and investigate the long-term impact of a transition clinic on medication adherence, health outcomes, and overall well-being among YPLHIV. Future interventions could include transition readiness assessments, transition protocols, use of mobile technology, and training and development of youth-friendly services in the adult HIV clinic.

## Figures and Tables

**Table 1:** Demographics of the AYAHCTC Population vs. Interviewed Patients.

	Eligible AYAHCTC Patients (n = 21)	Interviewed Patients (n = 5)
Sex		
Female	33.33%	0%
Male	66.66%	100%
Gender Identity		
Cisgender identity	100%	100%
Heterosexual	71.4%	60%
Bisexual	14.3%	20%
Homosexual	14.3%	20%
Race and Ethnicity		
Black race	85.7%	100%
Caucasian race	9.5%	0%
Asian race	4.8%	0%
Hispanic ethnicity	0%	0%
International Identity		
Internationally born	47.6%	40%
International adoptee	22.2%	20%
Primary Language		
English	90.5%	100%
Non-English	9.5%	0%
Insurance Status		
Private	61.9%	80%
Medicaid	38.1%	20%
HIV Transmission Group		
Non-perinatal	28.6%	20%
Perinatal	71.4%	80%

**Table 2:** Clinical Engagement in AYAHCTC.

Number of Patients	21
Mean age at 1st visit (years)	19.6
Average time in clinic (years)	2.21
Average number of visits	7.86
Visits per year (# visits/duration in clinic)	4.34
Engagement in first year (%)	100.0%
Retention in first year (%)	95.5%
Viral suppression at first visit (%)	66.7%
Viral suppression at last visit (%)	81.0%
Number of patients who transitioned out of AYAHCTC	11

**Table 3:** Summary of Survey Responses.

	Mean, SD
Social Provision Scale	14.1 +/− 2.0
HIV Stigma Scale	6.7 +/− 3.7
Transition Readiness Assessment Questionnaire	4.3 +/− 0.7
PHQ-9	2.0 +/− 1.2

**Table 4:** Self-Identified Patient-Specific Factors Affecting Transition.

Subthemes: Patient Contextual Factors	Selected Exemplary Quotes
Timing of HIV self-disclosure	I’ve always been taking medication […] I never knew why I was taking medication, until I turned maybe 10 and that’s when they told me. So, at that point, it was just like, “Okay.” It was never really a struggle living with HIV. […] It was just something I lived with.
	[Time of disclosure] would have been around eight or nine years of age.
Emotional adjustment to HIV status	It took years. It was just years of conversation from the adolescent clinic, from my provider that was at my children’s hospital. […] And when I say years, years, not two or three, this is like 10 years of conversations trying to get the guts and the courage to sit and talk about [my HIV status] and stop leaving it sitting in on me and it stressing me out. And it’s a lot of build up, anger and stuff like that.
Stigma	I don’t like saying [HIV], so you got to, excuse me. I’ll probably say ‘it’ or ‘status’. I guess dealing with that status, you kind of wonder if some people are really nice because they feel bad or they’re just being nice because they’re nice people. But I always like to think that they would be nice because they’re nice people, and that’s how I look at it.
	Just feeling like an outcast.
History with providers	I still keep in touch with [Dr. NAME at AYAHCTC] […] Very friendly staff, physicians very friendly, just makes you feel like you don’t have HIV to begin with.
Health literacy	The [laboratory] results, I personally don’t ever really check on until I go back to the office […] there’s a lot of things that I don’t want to worry myself about and they’re actually normal. So, I don’t even bother looking at my test results on my own, but I know that if I were, [Dr. NAME] is going to explain them to me, he’s going to explain them very thoroughly, he’s going to make an action plan with me right then and right there.

**Table 5:** Transition of Care Facilitators.

Facilitator	Exemplary Quote
Trust in AYAHCTC provider	And then the adolescents, [Dr. NAME at AYAHCTC] already knew [Dr. NAME at VCCC]. So, the fact that the two communicated made me more accepting of the transition.
Staff support in developing autonomy	My parents used to schedule all the appointments for me, but now before fully transitioning, [my providers] basically taught me, ‘Hey, you need to start doing this’, gave a little more responsibility, a real small step until I was fully able to do appointments by myself, order prescriptions myself. So, they slowly over a couple months or so were like ‘Hey, I’m going to start slowly not doing things for you’, and you slowly start picking up your own medication, which I got used to really quickly. And after that I was like, okay, I’ve been doing this for quite a while, I know what to do now.
Communication with AYAHCTC team	If I need something after the appointment that I forgot to talk to [Dr. NAME] about, I can ask my nurse and they’ll go get him. Or if he’s in another patient’s room, he’ll tell me, “Well, text me on the telehealth app” […] He’s always there.
	[Front desk staff] are always quick to respond. Even if they don’t answer, they may call me back in, I would say the longest probably was maybe an hour they’ll be calling back, but 9 times out of 10, they answer the phone.
	In the adolescent workup, yes, they prepared me for the next step, be like, ‘Hey, for the next step you’ll have to do X, Y, Z by yourself with little help from your parents’, which mentally prepared me like, okay, this is all for me and my health matters to me and my parents can intervene if need be, but this is all for me. So, it helped me get ready.
Feeling welcomed by the VCCC team	I met the physician that was going to be helping or taking care of me. I met the caseworkers, case managers, the front desk people. And it was more of a relief that everybody was very friendly towards [Dr. NAME from AYAHCTC] and they knew the whole situation. So, I guess it was relieving, not too scary.
AYAHCTC clinical environment	Whenever I walk in most doctors’ offices, dental offices, most places in the healthcare industry, they’re really overworked and overwhelmed at times, and you can definitely tell by the way that they greet you. It’s like they’re trying to get you in and out very quickly. With my adolescent clinic, I don’t really feel that way. I feel like whenever I’m walking in, they’re excited to see me.
	And I don’t feel like there’s judgment whenever I’m in [the waiting room], people are not staring at me. They don’t know why I’m going there because, I mean, it’s just an adolescent clinic, so it’s not like no one knows, oh, he has HIV or, oh, he’s done this, he’s done that. No, there’s not that pre-assumed, what’s the word, pre-assumed notion on a person. It’s just very calming, and I just feel like that’s where I should be.
	It could be intimidating to be in that [examination] room, and they make it less of that and more of a chill and comfort while doing the examination.
	I did like how every room has a privacy curtain. That was nice. Because I didn’t always feel like at any given moment, somebody could walk in, and I’d be exposed.
In-house phlebotomy	[The laboratory technologists] were very friendly. They were very, very good. They did their job quite well. And if I had a curious thought about why, what was the blood for, what’s the purpose of me getting the blood draw, they would tell me to get sample of this diagnosis, whatnot, and I’ll be at ease at that moment knowing that.
	[The phlebotomy room] is very comfortable. It’s very good. It gives distraction. When you look at the wall, you see various creatures, drawings on the wall. It’s a very chill environment. I like that.
	So, from pediatrics and adolescents, usually they have their own little spot for labs. When I went to the adult care clinic, I had to go to another spot for labs, which other people had to get labs. So, I was just used to being in and out. You do the examination, you do the lab in the same place, and then you’re out. And adults, it’s like you do the examination and you have to go downstairs and get your labs. And the labs, the waiting room or waiting area might take 40 min to wait.
Case management or healthcare navigator service	The [caseworker] gave me her card the first time I met her. She was like, “If you need anything, don’t hesitate. Call and let me know and we’ll figure it out together, or if I have to do some researching”. She’s there for her people. She’s not just going to leave you. It’s not the blind leading the blind.
	The [caseworker] will send me information on insurance policy plans, new prescriptions, and health plans. So, he’ll just keep me updated with new information surrounding about the healthcare of HIV, new medication, new testing that’s going on. He’ll text me at least once a month, making sure I’m good. So, he was a very friendly guy.
	My fixing [with HIV] was going to be within myself as far as getting comfortable with it […] rather than, “Hey, I want you to meet such and such, they’re going to be your case worker for the day”. And then they talk about it and it’s like, I don’t even know who you are. You haven’t been around. And I’m not even comfortable with telling close people. How do you know? It just didn’t feel comfortable for me to talk to them.
Counseling service	She was one who done kind of a little evaluation on me, and she was like, “You may be a little depressed. Do you want to go see someone?” And she was recommending all of these places for me to go and all of these people for me to see. […] I really do like that. It’s very sweet of her.
	I tried counseling, realized it was not for me. I didn’t like talking to people that hadn’t been through the same things and understood exactly what I was going through. It just felt very weird, and in a way, I felt a little judged going to someone.
Tour of the VCCC	[Dr. NAME] set up a whole tour with somebody who was in the adult care […] Let me get to know the people who are in the adult clinic, so the transition would be a little bit easier. So that was very helpful to me, as far as getting to know where I’m going, how it looks, instead of just being pushed into the unknown.
Data sharing	They already knew who I was and stuff when I got there […] I guess they transferred my file or something, but I don’t know.

**Table 6:** Transition of Care Barriers.

Barrier	Exemplary Quote
Loss of parental support	It was when I went to my first appointment by myself […] It was weird in a way since I’ve been only going with my parents and whatnot, so it was all up to me to listen and get the information and process it and choose the next option. So, I was usually the one to sit back and twiddle my thumbs while the parents talked to the doctor. But now just I was forced […] to listen and hear instead of playing around.
Loss of a trusted provider	For me, it’s not the fact that I have to transfer, it’s more so the fact that I have to have a new doctor, and I feel like I’ve built a bond with [Dr. NAME] already, and that what we have going on is something that’s working for us. And I really like that he’s starting to understand me, I’m starting to understand his expectations a lot more, and he just cares about his patients, so I want to see him [at the adult clinic]. My biggest challenge was switching physicians because like I said, at the time I was happy where I was at, happy with my care, built a really close relationship with my physicians and stuff like that. So, it’s always hard to leave when you build a really strong connection with people who are helping you.
Skepticism around adult HIV providers	I feel like a lot of adult doctors, they’re just there just to examine you and go on about their day. I like the conversations that me and [Dr. NAME] have, because sometimes I do go in there nervous, because I have very bad anxiety that I’m trying to work on, and he understands that. So, he talks to me, he explains everything. I feel like I’m not going to get that at other places at all.
Confusion about having multiple VCCC providers	[Dr. NAME at AYAHCTC] has my personal number, so we communicate through there. […] They’re switching out my [adult care] physician, so I never know what new physician I have. It’s usually on MyHealth.
Insufficient preparation to develop autonomy	I guess the beginning of the adult [care] […] just taught me you are responsible for your health and not anyone else. And that came as a shock in a way because I always relied on others to make choices for my health, but the fact that I’m in charge of making it is scary. In that small transition of adolescence to adult, it would’ve helped to learn how to reschedule things yourself and or set time for appointment.
Loss of confidentiality in the VCCC	When I was a kid, it was okay because everybody was in [AYAHCTC] for different reasons. But as an adult, it’s just kind of like, I don’t know, very uncomfortable because [VCCC is] in a whole separate part of the building, and it’s isolated. […] And everybody’s in there for the same thing. So, I feel like that takes the privacy away. So, I didn’t like that part. And that’s what took me so long to get out of the children’s part.
Perceived stigma in the VCCC	But with the VCCC, you go in there and I went to school with this person and there’s no other reason for you to be checking in over here but for that and I don’t know, it just felt weird. Now it’s one of those things where rumors can start, people accidentally saying something, “Oh, I seen such… How did you see him when you…” So, all that, it’s just so uncomfortable.

## Data Availability

The datasets presented in this article are not publicly available due to privacy and ethical restrictions. The datasets include sensitive information, such as the dates of birth of participants living with HIV, which cannot be disclosed to protect participant confidentiality. For more information, please contact the corresponding author.

## Supplementary Materials

The following supporting information can be downloaded at: https://www.mdpi.com/article/10.3390/tropicalmed9090198/s1, Supplementary File S1, Qualitative Interview Guide; Supplementary File S2, Codebook.
