# Supplementary material for: Mixed Methods Evaluation of a Youth-Friendly Clinic for Young People Living with HIV Transitioning from Pediatric Care
Source: Trop Med Infect Dis. 2024 Aug 28;9(9):198. doi: 10.3390/tropicalmed9090198 (PMC11435583; doi:10.3390/tropicalmed9090198)
Supplement: Supplementary file 1 [file tropicalmed-09-00198-s001.zip › Supplementary File S1 Qualitative Interview Questions.pdf]

**Supplementary File S1. Qualitative Interview Guide**

## Open Ended Questions

**Part 1: Clinical Visit**

| Very Poor | Poor | Fair | Good | Very good |
|---|---|---|---|---|
| 1 | 2 | 3 | 4 | 5 |

Please rate the following experiences:

**Waiting Room**

- Friendliness and courtesy of the front office staff
- Comfort of the waiting room

**Care Provider**

- Friendliness and courtesy of the care provider
- Comfort of the examination room
- Care provider's responses to your questions and concerns
- Care provider's efforts to include you in decisions about your treatment
- Amount of time provider spent with you
- Likelihood of your recommending this care provider to others

**Phlebotomy**

- Friendliness and courtesy of laboratory technologists
- Comfort of the phlebotomy room
- Timeliness of test results and explanations by your care provider

**Other Staff**

- Besides your care provider, who else did you interact with during your visit? (If participant needs examples: behavioral specialists, social workers)

Please rate the following experiences:

- Friendliness and courtesy of ____
- Likelihood of your recommending this service to others

**Clinic Services**

- What services did you find helpful during your visit? (If participant needs examples: STI testing and treatment, contraception and birth control services, case management, counseling services)
- What services did you find not as helpful?
- What other services do you wish were offered during your visit?

**Probe Questions**

Subsequent open-ended questions that may be asked include:

- What are some of your reasons for rating … very good/poor?
- What did you like/dislike about…?
- Can you give me an example of…? How did that make you feel?
- What makes you feel that way about…?
- Can you tell me more about that?

**Part 2: Communication with Care Provider**

- How do you usually communicate with your medical team (MyHealth, text, phone call)?
- What challenges do you have with reaching your medical team?

**Probe Questions**

Subsequent open-ended questions that may be asked include:

- Can you give me an example of…?
- How did that make you feel?
- Can you tell me more about that?
- What do you mean when you say…?

**Part 3: Other Clinic Sites**

- Do you interact with other community-based organizations for your medical care, such as Nashville Care, Oasis, or NICE?
- What services do you find helpful at those organizations?
- What are some services at those organizations that you wish were offered in your visits with Vanderbilt Pediatric HIV Clinic, Adolescent Transition Clinic, or VCCC?

**Probe Questions**

Subsequent open-ended questions that may be asked include:

- What did you find helpful about that service?
- Can you give me an example of…?
- Can you tell me more about that?
- What do you mean when you say…?

**Part 4: Transition of Care**

- What services did you find helpful during your transition of care changing from VPHC to Adolescent Transition Clinic?
- What services did you find helpful during your transition of care changing Adolescent Transition Clinic to VCCC?

(If participant needs examples: guided clinic tours, help with setting up first appointment, transportation services, meeting with case manager)

- What challenges did you experience during your transition of care changing from VPHC to Adolescent Transition Clinic?
- What challenges did you experience during your transition of care changing Adolescent Transition Clinic to VCCC?

**Probe Questions**

Subsequent open-ended questions that may be asked include:

- What did you find helpful about that service?
- Can you give me an example of…? How did that make you feel?
- How did that challenge impact you and your care?
- How could this clinic have helped you navigate that challenge?
- Can you tell me more about that?
- What do you mean when you say…?

**Part 5: Conclusion**

- Thank you for all of your responses, is there anything else you would like to add before we end?
